# Pediatric Intensive Care Core Outcomes–a modified Delphi consensus process (PIC-CO)

**DOI:** 10.1186/s13054-026-05878-1

**Published:** 2026-03-09

**Authors:** Nadine Mand, Victoria Lieftüchter, Jens H. Westhoff, Stefanie Hort, Richard Biedermann, Christine Müller-Brandes, Michael Merker, Hans Fuchs, Francesco Cardona, Angelina Beer, Florian Hoffmann, Frida Regner, Sarah Lampe, Cynthia Pönicke, Christoph Härtel, Christian Brickmann, Nora Bruns

**Affiliations:** 1 Neonatology and Pediatric Intensive Care Medicine, Department of Pediatrics, Marburg University, Marburg, Germany; 2 Pediatric Intensive Care Medicine, Dr. Von Hauner Children’s Hospital, Ludwig-Maximilians-University, LMU Munich, Munich, Germany; 3 Department I, Center for Pediatric and Adolescent Medicine, Medical Faculty Heidelberg, Heidelberg University, University Hospital Heidelberg, Heidelberg, Germany; 4 Pediatric Intensive Care Medicine, Department of Pediatrics With Focus On Pneumology, Immunology and Intensive Care Medicine, University Medicine Charité Berlin, Berlin, Germany; 5 Department of Pediatrics, University Hospital Jena, Jena, Germany; 6 Department of Anesthesiology, Pediatric Critical Care Medicine and Emergency Medicine, Children´S and Youth Hospital Auf Der Bult, Hanover, Germany; 7 Department for Children and Adolescents, Division for Neonatology, Critical Care, and Cardiology, Goethe University Frankfurt, Frankfurt (Main), Germany; 8 Neonatology and Ped. Critical Care, Center for Pediatrics, Medical Center, University of Freiburg, Freiburg, Germany; 9 Comprehensive Center for Pediatrics, Medical University of Vienna, Vienna, Austria; 10 Pediatric Intensive Care Medicine, Department of Pediatrics, University Clinic Carl Gustav Carus, TU Dresden, Dresden, Germany; 11 Department of Pediatrics, Klinikum Dritter Orden, Munich, Germany; 12 Department of Pediatrics, University of Würzburg, Würzburg, Germany; 13 TUM School of Medicine and Health, Department of Pediatrics, Kinderklinik Muenchen Schwabing, TUM University Hospital, Technical University of Munich, Munich, Germany; 14 Department of Pediatrics I, University Hospital Essen, University of Duisburg-Essen, Essen, Germany

**Keywords:** Critical Care / Standards, Outcome Assessment, Health care /Standards, Quality of Life / Psychology, Patient Reported Outcome Measures, Pediatric Intensive Care

## Abstract

**Purpose:**

Despite substantial improvements in survival after pediatric intensive care, long-term morbidity remains frequent and insufficiently assessed. No standardized, consensus-based core outcome set (COS) tailored to German-speaking regions exists. This study developed a Pediatric Intensive Care Core Outcome (PIC-CO) set to harmonize outcome assessment and follow-up of PICU survivors for clinical practice and research in German-speaking regions.

**Methods:**

A modified Delphi process was conducted within the Section of Pediatric Intensive Care and Emergency Medicine (SPIE) of the German Interdisciplinary Association of Intensive Care and Emergency Medicine. A multiprofessional expert panel identified outcome domains and questionnaires. Following internal consensus using predefined criteria, candidate questionnaires and short-term outcomes were submitted to an external Delphi survey distributed to SPIE members. Consensus and acceptance thresholds were defined a priori. Final questionnaire selection and recommendations for follow-up time points were agreed on by the expert panel.

**Results:**

Seventeen experts participated in the internal consensus phase. Of 65 evaluated questionnaires, 1–3 questionnaires per domain and 11 short-term outcomes were submitted to the external survey (46 respondents). All short-term outcomes and at least one questionnaire per domain reached acceptance; the majority achieved consensus. The final PIC-CO set comprises seven questionnaires across six domains and 11 short-term outcomes, alongside a structured follow-up schedule.

**Conclusions:**

The PIC-CO set provides the first consensus-based, pragmatically implementable COS for pediatric critical care in German-speaking countries, enabling standardized long-term assessment for clinical practice and research. Substantial overlap with international initiatives was observed, with differences mainly related to cognitive assessment and domain structuring.

**Supplementary Information:**

The online version contains supplementary material available at 10.1186/s13054-026-05878-1.

## Introduction

Survival rates in children and adolescents treated for critical illnesses in pediatric intensive care units (PICU) have significantly improved during the last decades [1]. Within the German Pediatric Intensive Care Unit Admissions (PIA) Network [2] the overall case fatality rate is less than 2% [3, 4], reflecting low mortality rates in high-income countries [5]. It is well-acknowledged that surviving children show increased rates of morbidity following a PICU stay, including functional and developmental deficits [1, 6], cognitive impairments, disturbances in family functioning, and decreased quality of life [7–10] in up to 70% of cases[1, 11]. Though an encouraging number of children recover, impairments frequently persist [12, 13]. 

Accordingly, pediatric critical care research increasingly focuses on the long-term effects on PICU survivors; nonetheless, outcome measures and time points for follow-up vary widely [1]. To address this, pediatric core outcome sets (COS) have been developed as standardized frameworks defining a minimum set of agreed-upon criteria for use in clinical trials to harmonize evidence synthesis, minimize research duplication and inconsistent reporting while increasing comparability of study results [14, 15]. However, most of the existing COS for children and adolescents are organ-specific and fail to comprehensively address the needs of PICU follow-up populations [14]. Furthermore, even systematically developed outcome sets and outcome measurement frameworks in pediatric intensive care have important limitations related to the intended care setting [6, 16, 17]. In the German healthcare system and worldwide, the lack of recommendations on the timing of follow-up [6] and the limited availability of remote or digital screening options to facilitate targeted on-site follow-up are among the most relevant limitations of current COS.

Consequently, structured follow-up after PICU survival is rarely performed, resulting in the prevalence of post-intensive care sequelae and pediatric post-intensive care syndrome (PICS-p) remaining largely under-researched, and persistently low awareness among pediatricians. Currently, no standardized COS tailored for implementation in the German healthcare system and agreed-upon by the pediatric intensive care community exists. This lack of standardized, locally endorsed outcome sets contributes to the ongoing heterogeneity of outcome assessment in both clinical practice and research. 

This project aimed to develop a COS for pediatric critical care to promote standardized and comparable outcome assessment across pediatric intensive care studies — within, but not limited to, German-speaking countries. It sought to encompass all dimensions of health that are crucial for long-term outcomes in children and adolescents to enhance the comparability of research findings, facilitate meta-analyses, and improve evidence synthesis in pediatric critical care outcomes research.

## Methods

This is a modified Delphi study that included formation of a multi-professional expert panel within the *Section of Pediatric Intensive Care and Emergency Medicine* (SPIE) of the *German Interdisciplinary Society of Intensive Care and Emergency Medicine* (DIVI), literature search and internal consensus on a set of questionnaires and items that were then submitted to an external Delphi survey with the SPIE and finally approved by the initial expert panel. This approach was chosen to integrate expert perspectives with community involvement to facilitate broad adoption.

### Formation of the expert panel

In September 2024, a retreat of the SPIE was held and the project was proposed to the participants of the retreat. All participants of the retreat were pediatric intensive care physicians or nurses with substantial practical experience in the field. A preliminary study group was formed, which presented the project proposal at the SPIE session of the annual meeting of the DIVI in December 2024 along with a call for collaboration. In December 2024 and January 2025, registrants were asked to confirm their intention to participate in the project. Of the 20 initial registrants, 17 (85%) provided confirmation.

### Literature search and identification of questionnaires

Domains to be addressed by the COS were identified from the literature [16, 18–20]. The subsequent PubMed search included “core outcome set” and domain specific key words for short- and long-term outcomes. From the initial set of identified questionnaires (Supplementary Table 1), those meeting the selection criteria were reviewed in more detail: Questionnaires should be well-established and internationally used with validated German translations available wherever possible. Further criteria were:

- free availability without license requirements.
- duration of assessment.
- compatibility with clinical follow-up.
- feasibility of digital or online administration, including the option for in-house programming (indicated as “online feasible” in the questionnaire).
- feasibility of remote assessment (patient- or parent-reported outcome measures).

### Short-term PICU outcomes

Short-term PICU outcomes were consented within the expert panel based on common outcomes in international pediatric critical care literature. Precautions were taken to ensure compatibility with the outcome list of the German nationwide pediatric intensive care registry (Pediatric Intensive Care Unit Admissions Network, PIA network) [2].

### Modified Delphi process

#### Internal consensus

Internal consensus on the questionnaires selected for submission to the external consensus was achieved via discussion within the panel. Out of a total of 65 identified questionnaires, each domain-specific team presented all potentially available questionnaires for their respective domain, including the questionnaire characteristics, advantages and disadvantages, and the team’s recommendation based on the selection criteria. The Pediatric Evaluation of Disability Inventory – Computer Adaptive Test (PEDI-CAT) was recommended for two domains and was counted as two questionnaires at this stage. For each domain, at least one and a maximum of three questionnaires were selected for the external consensus survey by the panel. For the short-term PICU outcomes, a total of 11 items was selected.

Before submitting the questionnaires and short-term PICU outcomes to the modified Delphi process, parents attending follow-up care at the University Hospital Würzburg were informally consulted regarding their judgement on the plausibility and feasibility of the selected questionnaires and items.

#### External consensus

The external consensus process consisted of an online survey conducted via LimeSurvey (Hamburg, Germany) that was sent out via email to members of the Pediatric Section of the German Interdisciplinary Association of Intensive Care and Emergency Medicine. The section has approximately 300 members. The questionnaires selected during the internal consensus process were presented including relevant information (Supplementary Material 1). The most appropriate questionnaire according to the internal consensus process was labeled as the recommended questionnaire. All questionnaires and items were to be rated on a 5-point Likert scale with „no answer” as an additional option. The scale ranged from 1 (completely disagree) through 5 (completely agree). At the end of each domain, a free-text field was provided for additional comments.

#### Definition of consensus and acceptance

Acceptance and consensus were conceptualized as related but distinct constructs reflecting different levels of agreement among panel members prior to the conduction of the survey. Acceptance was intended to capture the absence of substantive disagreement, whereas consensus represented a higher level of positive agreement.

Operationally, acceptance was defined as at least 80% of valid responses (excluding “no answer”) rating an item between 3 and 5, indicating that the item was not opposed by the majority of the respondents. Consensus was defined more stringently as at least 70% of valid responses rating the item 4 or 5, reflecting active agreement.

In this framework, consensus represents a more stringent criterion than acceptance, as it requires a clear majority of respondents to actively support an item rather than merely not disagree with it. 

#### Procedure for the final inclusion of items

The procedure for the final inclusion of items was defined prior to the conduction of the survey. One item or questionnaire was included from each domain, unless an exception had been defined in advance. If consensus or acceptance was reached on multiple items, the recommended questionnaire was included, otherwise the alternative questionnaire was to be included. Items that achieved neither consensus nor acceptance were to be excluded.

- Exception – PICU Outcomes: All items were recommended, and all items that reached consensus or acceptance were included.
- Exception – Cognitive Outcomes: Both the Pediatric Cerebral Performance Category (PCPC) and Pediatric Evaluation of Disability Inventory – Computer Adaptive Test (PEDI-CAT) were recommended by the study team.
- If no item within a domain was to reach consensus or acceptance:
  - The items in this domain were to be revised, and a new external consensus survey to be initiated. The community was to be consulted prior to the new consensus process to explore the reasons for the lack of acceptance.
  - A panel discussion was to be conducted to review and approve the final selection of questionnaires and items.

### Determining the timing of follow-up

Recommendations for the time points of follow-up were made based on current literature [7, 21–23] and current international practice within the PALISI network (Pediatric Acute Lung Injury and Sepsis Investigators) and at two of the participating centers (Ludwig-Maximilian-University Munich and University Hospital Essen) [24, 25].

### Competing interests 

No formal conflict-of-interest declarations were obtained from members of the expert panel or the validation cohort; however, as the study focused on outcome prioritization and involved no commercial interventions or industry sponsorship, the risk of relevant conflicts of interest was considered low.

### Statistical analysis

Data were descriptively analysed using RStudio Version 2025.09.0 + 387 (Posit Software, Boston, Massachusetts, USA).

## Results

**Fig. 1:**
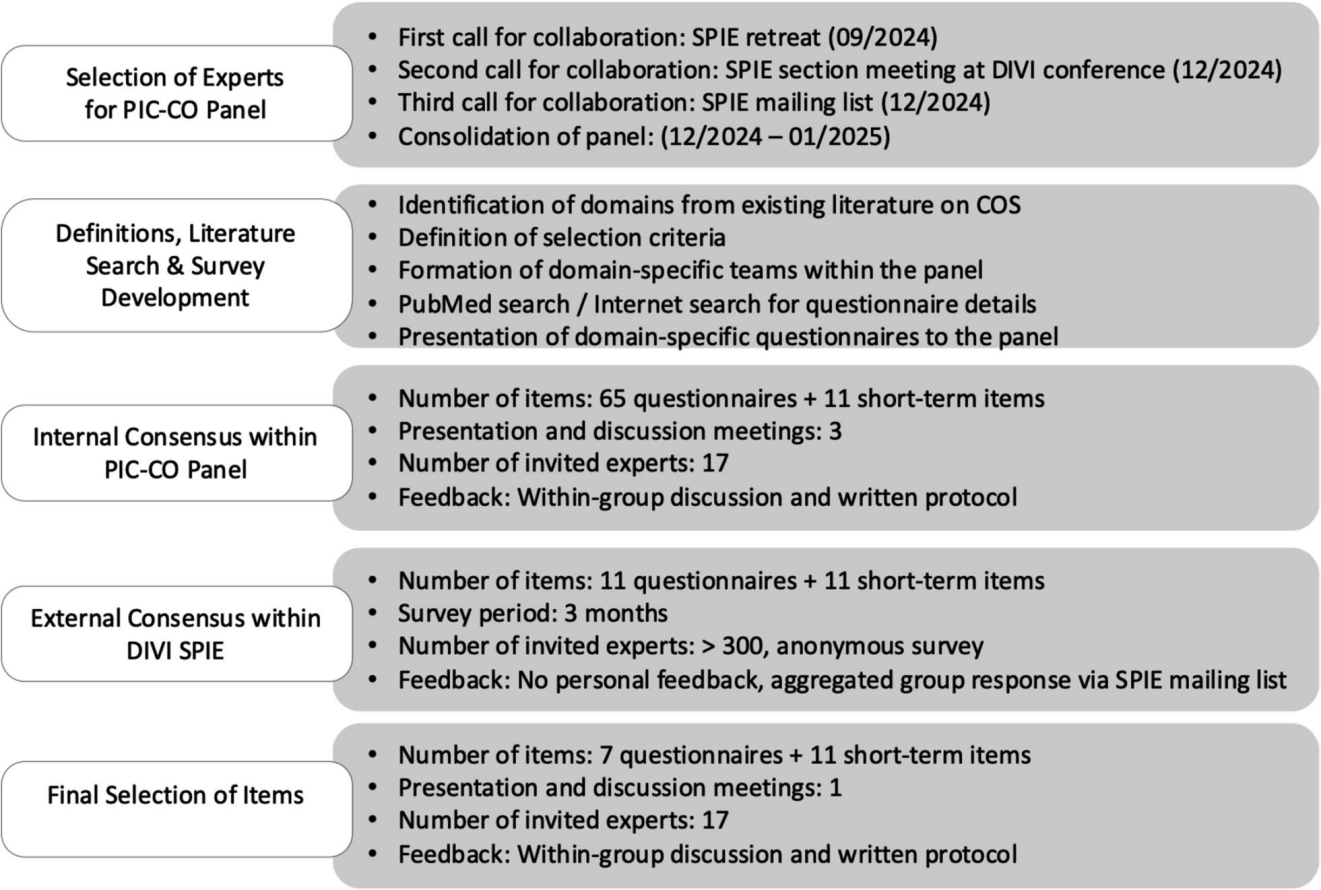
Delphi STAR flow chart—Steps of the modified Delphi process to identify a Pediatric Intensive Care Core Outcome (PIC-CO) Set. The number of invited experts for collaboration on PIC-CO set was > 300 and the number of experts that worked on definitions, literature search, and survey development was 17. COS – Core Outcome Set; DIVI – German Interdisciplinary Association of Intensive Care and Emergency Medicine; SPIE – Section of Pediatric Intensive Care and Emergency Medicine; DIVI – German Interdisciplinary Association of Intensive Care and Emergency Medicine

The panel for the internal consensus consisted of 17 experts (14 board-certified pediatricians with subspecialty board certification in pediatric intensive care, 2 psychologists, 1 nurse with board certification in pediatric intensive care), of whom 9 (53%) were female (Fig. 1).

### Internal consensus

Six domains (physical function, cognitive function, emotion (divided into post traumatic stress disorder/behaviour and anxiety/depression), quality of life, and family function)232 were identified, with short-term PICU outcomes as an additional section. For each domain, between one and three questionnaires fulfilled the suitability criteria. Suitable questionnaires and short-term PICU outcomes were submitted to the external consensus survey with unanimous approval by the panel (17/17 votes) (Table 1).

**Table 1:** Pediatric Intensive Care Core Outcome (PIC-CO) Set recommended by the Pediatric Section of the German Interdisciplinary Association of Intensive Care and Emergency Medicine

Short-term PICU outcomes	Questionnaires
• All-cause in-hospital mortality • All-cause 30-day-mortality • All-cause 90-day-mortality • Duration of mechanical ventilation (Time from intubation to successful extubation → absence of mechanical ventilation for 48 consecutive hrs) [days] • Duration of continuos sedation by infusion (including sevoflurane/isoflurane) [days] • Duration of vasopressor/inotrope administration • Length of stay ICU [days] • Length of stay hospital [days] • Discharge destination (home, rehabilitation, other hospital, nursing facility) • PCPC at discharge and PCPC decline (defined as difference between PCPC discharge and pre-illness PCPC before current PICU admission) • Newly established devices: o trachostomy o PEG/PEJ/jejunal tube or nasogastric tube as bridging until PEG/PEJ o Respiratory support* (oxygen, high-flow nasal cannula, non-invasive ventilation or invasive ventilation) o Dialysis	• Domain 1—physical function: o Pediatric evaluation of disability inventory – computer adaptive test (PEDI-CAT) [34] • Domain 2—cognitive function: o Pediatric cerebral performance category (PCPC) [35] o Pediatric evaluation of disability inventory – computer adaptive test (PEDI-CAT) [34] • Domain 3—emotion – post-traumatic stress-disorder (PTSD) and behaviour: o Child and adolescent trauma screening (CATS) [36] • Domain 4—emotion—anxiety/ depression: o Revised child anxiety and depression scale 25 (RCADS 25) [37] • Domain 5—quality of life, participation, social relationships, mental health: o Strength and difficulties questionnaire (SDQ) [38] o Pediatric quality of life inventory / infant scales (PedsQL) [39] • Domain 6—family function: o Impact on family scale (FaBel) [27]

^*^newly established or escalated compared to pre-illness support

### External consensus

Fifty-two surveys (Supplementary Material 1) were clicked through until the end in the survey tool, of which 46 received at least one answer. Only the 46 surveys with at least one answer were included in the statistical analysis (Tables 2 and 3). Answers were received from physicians (n = 43; 93%), psychologists (n = 2; 4%) and one nurse (2%).

**Table 2:** Survey results for domains 1 through 6

Domain	Survey	Valid answers	Missing	Response rate (%)	Median score	Counts for consensus*	Counts for acceptance**
1: Physical function	PEDI-CAT	42	4	91.3	4.5	29 (69.0%)	37 (88.1%)
2: Cognitive function	PCPC	44	2	95.7	5	36 (81.8%)	41 (93.2%)
	PEDI-CAT	44	2	95.7	4	30 (68.2%)	38 (86.4%)
3: Emotion—PTBS and behaviour	CATS	42	4	91.3	5	33 (78.6%)	40 (95.2%)
	CRIES-8	38	8	82.6	3	16 (42.1%)	28 (73.7%)
4: Emotion—anxiety/ depression	CADS 25	44	2	95.7	5	36 (81.8%)	43 (97.7%)
	CADS 47	35	11	76.1	3	16 (45.7%)	27 (77.1%)
	CBCL	35	11	76.1	3	13 (37.1%)	21 (60.0%)
5: Quality of life, participation, social relationships, mental health	SDQ	41	5	89.1	5	34 (82.9%)	39 (95.1%)
	PedsQL	39	7	84.8	4	24 (61.5%)	33 (84.6%)
6: Family function	FaBel	44	2	95.7	5	39 (88.6%)	43 (97.7%)

Number of analysed surveys: 46; * threshold: 70%; ** threshold: 80%; percentages for consensus and acceptance refer to the number of valid answers as denominator

**Table 3:** Results for short-term PICU outcomes

Item	Valid answers	Missing	Response rate (%)	Median score	Counts for consensus*	Counts for acceptance**
All-cause in-hospital mortality	40	6	87.0	5	37 (92.5%)	38 (95.0%)
All-cause 30-day-mortality	39	7	84.4	5	32 (82.1%)	37 (94.9%)
All-cause 90-day-mortality	8	8	82.6	5	32 (84.2%)	36 (94.7%)
Duration of invasive mechanical ventilation	41	5	89.1	5	39 (95.1%)	41 (100%)
Duration of continuous sedation by infusion	39	7	84.8	5	36 (92.3%)	38 (97.4%)
Duration of vasopressor/inotrope administration	39	7	84.8	5	35 (89.7%)	38 (97.4%)
Length of stay ICU	40	6	87.0	5	38 (95%)	39 (97.5%)
Length of stay hospital	39	7	84.8	5	36 (92.3%)	37 (94.9%)
Discharge destination	40	6	87.0	5	35 (87.5%)	38 (95.0%)
PCPC at hospital discharge and PCPC decline with respect to pre-illness score	37	9	80.4	5	34 (91.9%)	36 (97.3%)
Newly established devices at hospital discharge	38	8	82.6	5	35 (92.1%)	37 (97.4%)

Number of analysed surveys: 46; * threshold: 70%; ** threshold: 80%; percentages for consensus and acceptance refer to the number of valid answers as denominator

All questionnaires but three achieved acceptance: CRIES-8 (domain 3) and CADS-47 and CBCL (both domain 4) were not accepted (Table 2). Two questionnaires that achieved acceptance did not achieve consensus: PEDI-CAT (domains 1 and 2) and PedsQL (domain 5) (Table 2). All short-term PICU outcomes achieved acceptance and consensus (Table 3). Overall, at least one questionnaire from each domain achieved acceptance, and except for domain 1, at least one questionnaire from each domain achieved consensus.

### Final selection of items

The final selection of items followed the a priori-defined procedure that is detailed in the methods section. However, the board discussion revealed that both the SDQ and the PedsQL should have been recommended in the online Delphi survey, as they cover different areas with the domain *„*Q*uality of Life, Participation, Social Relationships, Mental Health”*. Because both questionnaires achieved acceptance and the SDQ achieved consensus additionally, the board decided unanimously to include both questionnaires in the final selection of seven recommended items. The PEDI-CAT is recommended for two domains (physical function and cognitive function) but is counted as one assessment. All short-term PICU outcomes were included into the final recommendation. The final selection of questionnaires was approved unanimously (17/17) by the panel.

### Recommendation for time points of follow-up

**Fig. 2:**
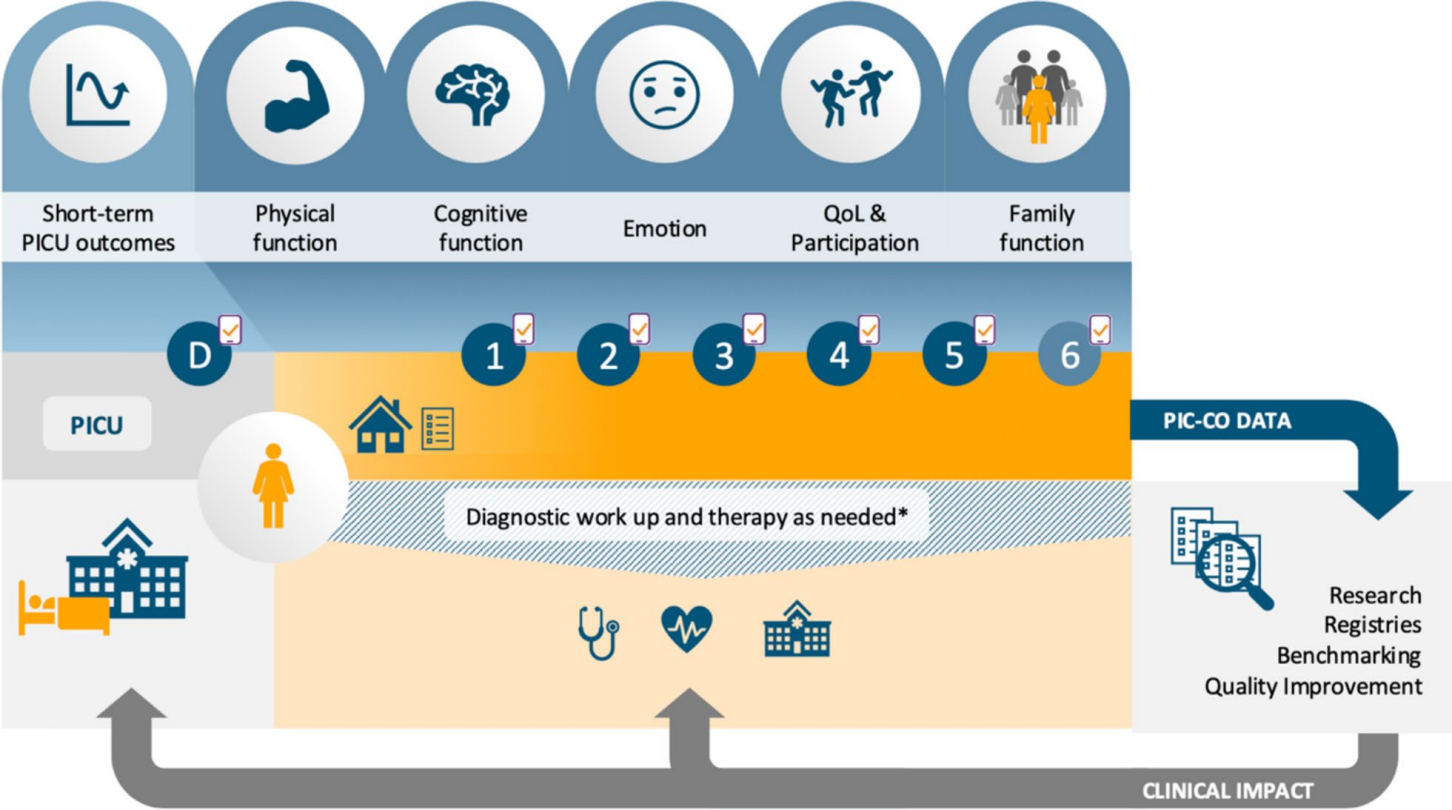
Pediatric Intensive Care Core Outcome (PIC-CO) Set and its possible integration into clinical practice and research. QoL – Quality of Life. *in outpatient department or via inpatient admission

Based on the literature [7, 21–23], the experience from two centers participating in this study showing feasibility [24, 25], and international practice, our panel recommends the following time points mended for follow-up (Fig. 2):

- Time point 0: Pre-illness (retrospective assessment)
- Time point 1: Pre-discharge
- Time point 2: 4 – 6 weeks post discharge
- Time point 3: 3 months post discharge
- Time point 4: 6 months post discharge
- Time point 5: 12 months post discharge
- Optional: Time point 6: 24 months post discharge
- On-site visits as per local standard (additionally or complementary) and any time if clinically indicated

Not all tests and questionnaires are appropriate, necessary, or feasible at all time points; their application should therefore be guided by structured institutional protocols adapted to local prerequisites.

## Discussion

Core outcome sets in general and particularly in critically ill children are essential to assess the impact of medical interventions on outcomes. The provision of value-based pediatric critical care requires outcome measures that reflect what is meaningful to children and their families, capturing a multidimensional perspective. In research, the precise definition and measurement of study endpoints is critical for determining sample sizes, conducting systematic reviews, and establishing guidelines that subsequently influence clinical practices and health policies.

In this modified Delphi process, we established the PIC-CO set— a core minimum set of short- and long-term outcomes suitable for implementation in German-speaking post-PICU care settings. The set covers survival, resolution of organ dysfunction, and key long-term domains, thereby supporting a holistic and family-centered approach to follow-up in a growing and heterogeneous population of PICU survivors. The longitudinal, follow-up addresses not only safety aspects [23, 26] but also provides an important tool to inform benchmarking and develop new hypotheses for protective interventions, redirection of care, and tertiary prevention. Outcome and instrument selection was guided by existing evidence and expert consensus, with a focus on validity, feasibility, and pragmatic implementation. Emphasis on accessibility and integration into routine and digital follow-up structures supports sustainable use while minimizing the burden for families and health care providers. In the final PIC-CO set, all questionnaires except for the PEDI-CAT are cost-free and the FaBel is the only questionnaire that is not available in multiple languages. However, the FaBel is the German validated translation of the Impact on Family Scale [27, 28], making it internationally applicable.

Compared with previous initiatives [6, 16], there is substantial overlap in the recommended instruments. However, earlier frameworks typically allow greater flexibility by offering multiple options per domain and distinguishing between core and extended assessments, whereas the PIC-CO set provides more explicit guidance to support standardized implementation. Importantly, all instruments recommended in the PIC-CO set are also included in these broader international recommendations. Meaningful differences are largely confined to the cognitive and social domains. While previous initiatives recommend NIH Toolbox or PROMIS instruments, the PIC-CO set prioritizes PCPC and PEDI-CAT for cognitive assessment. In addition, the social domain which is recommended to be assessed using NIH Toolbox instruments [6] —is not defined as a separate domain in the PIC-CO set. Nevertheless, the underlying instruments across domains largely overlap, and these differences primarily reflect variations in domain structuring rather than divergent approaches to outcome assessment.

The finalized set allows comparison between units and heterogeneous populations of critically ill children, standardizes research and facilitates inclusion of children in adaptive trials of critical care interventions across all age groups. It provides a continuum into follow-up that is compatible with the PIA network item list for PICU stay [29]. Notably, the PIC-CO set considers the affected child, their family and social context as interacting determinants of long-term health [30], allowing adjustment for individual risk factors of the child (e.g., age and pre-existing morbidity), family factors (e.g., distress and socioeconomic status), in addition to broadly-established illness/treatment factors such as scores to quantify the severity of disease that should be applied. An international two-round Delphi study [16], recommended a PICU COS for pediatric critical care with four global domains (cognitive, emotional, physical and global health) and four specific domains (child-health related quality of life, pain, survival, and communication). In contrast to our approach that included experts from Germany and Austria, that study’s major strength was the involvement of multiple stakeholder groups, including families and advocates. Our work adds a regionally adapted, implementable set tailored to the German healthcare context, which complements existing international efforts. Even though stakeholder representation was limited in this study, the identified outcomes are in line with previous studies that identified preferred outcomes of PICU family care providers and health care professional [31–33]. In addition to survival, these studies’ results emphasize organ and physiological functioning, duration of invasive mechanical ventilation and PICU stay, as well as longer-term quality of life and functional status. To address our limited stakeholder representation including the small number of involved clinical psychologists and lack of structured patient/family involvement, we are planning a qualitative study that will comprise PICU survivors, their families, and multiprofessional healthcare providers to evaluate the PIC-CO set, identify potential barriers for its implementation, and strategies to overcome these limitations. We also plan to participate in an observational study of the Study Network Critical Care of the German Network University Medicine for pilot testing of feasibility in a multicenter context.

A further limitation is the development of the set solely by experts from German-speaking regions. Hence, international generalizability may be limited, and not all recommended questionnaires are available or validated in other languages. Importantly, the PIC-CO set represents a core outcome *minimum* dataset. It is not intended to cover all aspects of long-term morbidity comprehensively, but rather to ensure that a standardized baseline of essential domains is captured across studies and clinical programs. Additional assessments, including anthropometric measures or disease-specific evaluations, may need to be incorporated as appropriate to the research question or clinical context. Future releases may further emphasize congruence with international practice by incorporating additional, more granular functional outcome instruments (e.g., the Functional Status Scale) to enhance comparability across initiatives and improve clinical follow-up. In its current form, the PIC-CO set primarily functions as a screening tool where positive findings should trigger a structured, domain-specific diagnostic work-up and referral to specialized services if needed. Some of the selected questionnaires have limited applicability in children with pre-existing developmental disabilities, particularly those relying on child self-report or higher cognitive functioning. In these cases, results should be interpreted with caution and, where appropriate, complemented by parent-reported or functional measures.

The strengths of our approach lie in the identification of a feasible, consensus-based COS for pediatric critical care, which supports harmonized outcome assessment in Germany and enables comparative research and structured long-term monitoring. By explicitly supporting digital and remote assessment, the PIC-CO set expands opportunities for scalable follow-up in studies and routine healthcare and can be integrated flexibly into workflows and care pathways, and offers the potential for implementation even in low-resource settings. Other health-related outcomes, such as unmet needs and hospitalization rates, and process outcomes, such as professional education initiatives, cost evaluations and resource utilization, may be integrated into this framework. Involving additional stakeholders will enhance broad adoption of this framework, promote research into pediatric critical care outcomes, foster international collaboration, and improve clinical care. Ultimately, the PIC-CO set represents an important step towards improving long-term survivorship and care quality for critically ill children.

## Supplementary Information

Supplementary Material 1

Supplementary Material 2

## Data Availability

The datasets used and/or analysed during the current study are available from the corresponding author on reasonable request.

## Abbreviations

CADS-47: Children’s anxiety and depression scale
CBCL: Child behavior checklist
COS: Core outcome data set
CRIES-8: Child revised impact of event scale
DIVI: German interdisciplinary society of intensive care and emergency medicine
PCPC: Pediatric cerebral performance category
PEDI-CAT: Pediatric evaluation of disability inventory – computer adaptive test
PedsQL: Pediatric quality of life inventory
PICS-p: Pediatric post-intensive care syndrome
PICU: Pediatric intensive care unit
PIA: Pediatric intensive care unit admissions network
PIC-CO: Pediatric intensive care core outcomes
SDQ: Strengths and difficulties questionnaire
SPIE: Section of pediatric intensive care and emergency medicine of the German Interdisciplinary Association of Intensive Care and Emergency Medicine (DIVI)
