# Supplementary material for: Pediatric Intensive Care Core Outcomes–a modified Delphi consensus process (PIC-CO)
Source: Crit Care. 2026 Mar 9;30:160. doi: 10.1186/s13054-026-05878-1 (PMC13064096; doi:10.1186/s13054-026-05878-1)
Supplement: Supplementary file 2 — Supplementary Material 2 [file 13054_2026_5878_MOESM2_ESM.docx]

Pediatric Intensive Care Core Outcomes - Delphi Survey

Dear colleagues,

In the following survey, we would like to present you with proposals for **Pediatric Intensive Care Core Outcomes**. This is a project of the Research Working Group of the DIVI Section **Pediatric Intensive and Emergency Medicine**, with the goal of defining a toolkit of relevant clinical outcome parameters.

These core outcomes are intended to establish common standards to enable better comparison of future clinical studies. Some categories are also relevant for clinical follow-up. In each category, several items are provided for selection.

All proposed questionnaires or items are assessed using a **5-point Likert scale**. You do **not** need to choose between the (usually 2) proposed items; instead, you can indicate your degree of agreement for each item individually. The questionnaire identified by our group as the most suitable after extensive research is marked accordingly.

If you cannot or do not wish to assess an item, please leave the selection at the default **"no answer"**.

The focus in selecting the proposed items was on:

- Free availability without licensing
- Duration of administration
- Compatibility with clinical follow-up
- Possibility of digital/online administration, possibly through custom programming (marked in the survey as **"online possible"**)
- Possibility of **remote** administration (patient/parent-reported outcome measures)

If you have additional suggestions or comments, there is a free-text field at the end of each category.

Thank you very much for your support!

This survey contains **15 questions**.

# **1. Professional category**

Which professional group do you belong to? Please select one of the following answers:

- Physician / Medical Staff
- Nurse / Nursing Staff
- Psychologist
- Other

# **2. Physical function**

Please indicate the degree of your agreement for the proposed items. If you cannot assess an item, please select "no answer." Please select the appropriate answer for each item:

|  | ***1 – do not agree*** | ***2*** | ***3*** | ***4*** | ***5 – fully agree*** | ***No answer*** |
| --- | --- | --- | --- | --- | --- | --- |
| *PEDI-CAT (recommended)* |  |  |  |  |  |  |

Explanation of the proposed items:

***PEDI-CAT – Pediatric Evaluation of Disability Inventory – Computer Adaptive Test*:**

- Covered age groups: 6 months to 20 years
- Parent questionnaire (PROM)
- Multilingual
- 2.05 EUR/test, research license possible
- Online administration + evaluation, no training required
- Duration: 10-15 min for all categories
- Categories:
  - Activities of daily living
  - Mobility
  - Social and cognitive skills
  - Responsibility
- Cross-reference: recommended for physical functioning and cognition

Comments:

Please enter your answer here:

|  |
| --- |

# **3. Cognitive function**

Please indicate the degree of your agreement for the proposed items. If you cannot assess an item, please select "no answer." Please select the appropriate answer for each item:

|  | ***1 – do not agree*** | ***2*** | ***3*** | ***4*** | ***5 – fully agree*** | ***No answer*** |
| --- | --- | --- | --- | --- | --- | --- |
| *PCPC (recommended)* |  |  |  |  |  |  |
| *PEDI-CAT (recommended)* |  |  |  |  |  |  |

Explanation of the proposed items:

**PCPC – Pediatric Cerebral Performance Category**

- Covers all age groups
- Free of charge
- No specialized training required
- Duration < 5 min, only rough screening
- Can be done online
- 5 categories + death
- Cross-reference: also included in PICU Outcomes for statistics, internationally recognized and widely used, can also be collected retrospectively from records

**PEDI-CAT – Pediatric Evaluation of Disability Inventory – Computer Adaptive Test**

- Covered age groups: 6 months to 20 years
- Parent questionnaire (PROM)
- Multilingual
- 2.05 EUR/test, research license possible
- Online administration + evaluation, no training required
- Duration: 10–15 min for all categories
- Categories:
  - Activities of daily living
  - Mobility
  - Social and cognitive skills
  - Responsibility
- Cross-reference: recommended for physical functioning and cognition

Comments:

Please enter your answer here:

|  |
| --- |

# **4. Emotion - Post-traumatic stress disorder and behavior**

Please indicate the degree of your agreement for the proposed items. If you cannot assess an item, please select "no answer." Please select the appropriate answer for each item:

|  | ***1 – do not agree*** | ***2*** | ***3*** | ***4*** | ***5 – fully agree*** | ***No answer*** |
| --- | --- | --- | --- | --- | --- | --- |
| *CATS (recommended)* |  |  |  |  |  |  |
| *CRIES-8* |  |  |  |  |  |  |

Explanation of the proposed items: This question group includes 2 screening questionnaires for post-traumatic stress disorder

**CATS - Child and Adolescent Trauma Screening**

- From 3 years
- Parent questionnaire (PROM)
- Free of charge
- German version (+ other languages)
- Can be done online
- Duration: 10–15 min
- From 3 years: third-party report; from 7 years: self-report possible
- Categories:
  - Checklist of traumatic events
  - Symptom questions
  - Functional impairment
  - Behavior

**CRIES-8 - Children's Impact of Event Scale**

- From 8 years
- Parent questionnaire (PROM)
- Free of charge
- German version (+ 28 languages)
- Can be done online
- Duration: 5 min
- Categories:
  - Intrusion
  - Avoidance

Comments:

Please enter your answer here:

|  |
| --- |

# **5. Emotion - Anxiety and Depression**

Please indicate the degree of your agreement for the proposed items. If you cannot assess an item, please select "no answer." Please select the appropriate answer for each item:

|  | ***1 – do not agree*** | ***2*** | ***3*** | ***4*** | ***5 – fully agree*** | ***No answer*** |
| --- | --- | --- | --- | --- | --- | --- |
| *RCADS-25 (recommended)* |  |  |  |  |  |  |
| *RCADS-47* |  |  |  |  |  |  |
| *CBCL* |  |  |  |  |  |  |

Explanation of the proposed items:

**RCADS 25/47 - Revised Child Anxiety and Depression Scale**

- From 6 years
- Parent questionnaire (PROM)
- Validated German translation
- Can be done online
- Free of charge
- Duration: 5–10 min for short version (RCADS-25) and 15–20 min for long version (RCADS-47)
- Categories:
  - Depression
  - Anxiety
  - Behavior
  - Somatic complaints

**CBCL - Child Behaviour Checklist**

- From 1.5 years
- Parent questionnaire (PROM)
- Partially validated German translation
- Online availability unclear
- Paid: €300 base package, then €20/25 questionnaires; research license possible
- Duration: 15–20 min
- Categories:
  - Behavior
  - Somatic complaints
  - Social competencies
  - Emotions

Comments:

Please enter your answer here:

|  |
| --- |

# **6. Quality of life, Participation, Social Relationships, Mental Health**

Please indicate the degree of your agreement for the proposed items. If you cannot assess an item, please select "no answer." Please select the appropriate answer for each item:

|  | ***1 – do not agree*** | ***2*** | ***3*** | ***4*** | ***5 – fully agree*** | ***No answer*** |
| --- | --- | --- | --- | --- | --- | --- |
| *SDQ (recommended)* |  |  |  |  |  |  |
| *PedsQL* |  |  |  |  |  |  |

Explanation of the proposed items:

**SDQ - Strengths and Difficulties Questionnaire**

- From 2 years
- Parent questionnaire (PROM)
- Validated German translation
- Free of charge
- Can be done online
- Duration: 5–10 min
- Categories:
  - Emotional problems
  - Behavioral problems
  - Hyperactivity/inattention
  - Peer relationship problems
  - Prosocial behavior

**PedsQL - Pediatric Quality of Life Inventory / PedsQL Infant Scales**

- From 2 years or from 1 month (infant scales)
- Parent questionnaire (PROM)
- Multilingual
- Validated
- Free for research without funding
- Can be done online
- Categories:
  - Physical functioning
  - Emotional functioning
  - Social functioning
  - School functioning (Infant: cognitive functions)
  - Additional disease-specific modules

Comments:

Please enter your answer here:

|  |
| --- |

# **7. Family function**

Please indicate the degree of your agreement for the proposed items. If you cannot assess an item, please select "no answer." Please select the appropriate answer for each item:

|  | ***1 – do not agree*** | ***2*** | ***3*** | ***4*** | ***5 – fully agree*** | ***No answer*** |
| --- | --- | --- | --- | --- | --- | --- |
| *FaBel (recommended)* |  |  |  |  |  |  |

Explanation of the proposed items:

**FaBel - Impact on Family Scale / Family Burden Questionnaire**

- Covers all age groups
- Parent questionnaire (PROM)
- Free of charge
- Validated German version
- Duration: 10–15 min
- Categories:
  - Daily social burden
  - Personal burden/concerns about the future
  - Financial burden
  - Burden on siblings
  - Coping difficulties

Comments:

Please enter your answer here:

|  |
| --- |

# **8. Short-term PICU outcomes**

Please indicate the degree of your agreement for the proposed items. If you cannot assess an item, please select "no answer." Please select the appropriate answer for each item:

|  | ***1 – do not agree*** | ***2*** | ***3*** | ***4*** | ***5 – fully agree*** | ***No answer*** |
| --- | --- | --- | --- | --- | --- | --- |
| *All-cause in-hospital mortality* |  |  |  |  |  |  |
| *All-cause 30-day-mortality* |  |  |  |  |  |  |
| *All-cause 90-day-mortality* |  |  |  |  |  |  |
| *Duration of invasive mechanical ventilation [days]* |  |  |  |  |  |  |
| *Duration of continuos sedation via syringe pump (including sevoflurane/isoflurane) [days]* |  |  |  |  |  |  |
| *Duration of vasopressor/inotrope administration* |  |  |  |  |  |  |
| *Length of stay ICU [days]* |  |  |  |  |  |  |
| *Length of stay hospital [days]* |  |  |  |  |  |  |
| *Discharge destination* |  |  |  |  |  |  |
| *PCPC at hospital discharge and PCPC decline* |  |  |  |  |  |  |
| *Newly established devices at hospital discharge* |  |  |  |  |  |  |

**Important:** The items included in this list are **NOT** in competition with each other. Every item with sufficiently high agreement values will be included.

**Explanation of the proposed items:**

This question group includes relevant outcomes after PICU, which can primarily be used for publications. All items except two are measured on a continuous scale; the proposed unit is indicated in square brackets, e.g., [days].

**Duration of mechanical ventilation** is defined as “Time from intubation to successful extubation —> absence of invasive mechanical ventilation for 48 consecutive hours”

**PCPC decline** is defined as the difference between PCPC before admission and PCPC at discharge

**Discharge destination** – the question includes the following categories for single selection:

- Home
- Rehabilitation
- Other hospital
- Nursing facility

**Newly established devices at hospital discharge** – the question includes the following categories for multiple selection:

- Tracheostomy
- PEG/PEJ/jejunal tube or nasogastric tube as bridging until PEG/PEJ
- Respiratory support (oxygen, high-flow nasal cannula, non-invasive ventilation, invasive ventilation, cough assist, Mini-Pegaso)
- Dialysis

Comments:

Please enter your answer here:

|  |
| --- |
